# Flexible and Expedited Regulatory Review Processes for Innovative Medicines and Regenerative Medical Products in the US, the EU, and Japan

**DOI:** 10.3390/ijms20153801

**Published:** 2019-08-03

**Authors:** Sumimasa Nagai

**Affiliations:** Translational Research Center, The University of Tokyo Hospital, 7-3-1, Hongo, Bunkyo-ku, Tokyo 113-8655, Japan; sunagai-tky@umin.ac.jp; Tel.: +81-3-5800-9072

**Keywords:** EMA, FDA, PMDA, conditional approval, accelerated approval, Sakigake, breakthrough, PRIME, RMAT

## Abstract

Several expedited regulatory review projects for innovative drugs and regenerative medical products have been developed in the US, the EU, and Japan. Each regulatory agency has elaborated an original regulatory framework and adopted regulatory projects developed by the other regulatory agencies. For example, the Food and Drug Administration (FDA) first developed the breakthrough therapy designation, and then the Pharmaceuticals and Medical Devices Agency (PMDA) and European Medicines Agency (EMA) introduced the Sakigake designation and the priority medicines (PRIME) designation, respectively. In addition, the necessity of the product being first development in Japan is the original feature of the Sakigake designation, while actively supporting the development of advanced-therapy medicinal products (ATMPs) by academia or small/medium-sized sponsors is the original feature of the PRIME; these particular features are different from the breakthrough therapy designation in the US. In this review article, flexible and expedited review processes for new drugs, and cell and gene therapies in the US, the EU, and Japan are described. Moreover, all the drugs and regenerative medical products that were granted conditional approval or Sakigake designation in Japan are listed and analyzed herein.

## 1. Introduction

In this review article, flexible and expedited review processes for new drugs and cell and gene therapies in the US, the EU, and Japan are described. Regulations for medical devices and in vitro diagnostics (IVD) are not mentioned.

In the US, the Food and Drug Administration (FDA) reviews marketing authorization applications and approves medical devices, drugs, and regenerative medical products. The Center for Drug Evaluation and Research at the FDA is responsible for reviewing biologics license applications (BLA) for biosimilars and new antibodies, as well as new drug applications (NDA). The Center for Biologics Evaluation and Research is responsible for reviewing BLAs for gene and cellular therapy. Labels, review reports, and approval letters of medical products granted marketing authorization are publicly available on the FDA’s website [1,2]. As of 31 May 2019, 17 regenerative medical products approved by the FDA are listed on the website [2].

The European Medicines Agency (EMA) deals with the centralized authorization procedure of medicines and regenerative medical products in the EU. The centralized authorization procedure is compulsory for drugs for acquired immunodeficiency syndrome, diabetes, cancer, immune dysfunctions, and neurodegenerative diseases, as well as biosimilar products, advanced-therapy medicinal products (ATMPs), genetically engineered products, and orphan drugs [3,4,5]. ATMPs include gene-therapy medicines, cellular and gene therapy products, and tissue-engineered products [6]. A rapporteur and co-rapporteur appointed by the Committee for Medicinal Products for Human Use (CHMP) and/or the Pharmacovigilance Risk Assessment Committee review applications through the centralized authorization procedure [7]. The Committee for Advanced Therapies (CAT) at the EMA is responsible for assessing the ATMP [6]. Packaging inserts and review reports (public assessment reports) of approved drugs and ATMPs as well as withdrawn applications are listed on the EMA website [8].

The Pharmaceuticals and Medical Devices Agency (PMDA) reviews applications for drugs, medical devices, and regenerative medicines, and prepares review reports in Japan. The Ministry of Health, Labor and Welfare (MHLW) grants marketing authorization. The MHLW revised the Pharmaceutical Affairs Law and implemented the Law on Securing Quality, Efficacy and Safety of Products, including the Pharmaceuticals and Medical Devices Act in 2014 [4,9,10]. “Regenerative medical products” were newly defined in the Act as follows: (1) Products used by introducing them into human cells for gene therapy; and (2) processed cells used for the reconstruction/repair/formulation of human body structure/function or treatment/prevention of a disease. Regenerative medical products include vivo and ex vivo gene medical products and cellular medical products. Organ transplantation, hematopoietic stem cell transplantation, and blood transfusion are not classified as regenerative medical products [4,9,10]. All new drug/regenerative medical product applications are submitted to the PMDA. The offices of new drugs I–V, the office of vaccine and blood products, and the office of cellular and tissue-based products deal with new drugs and regenerative medical products [11]. The office of new drugs I mainly handles medicines to treat gastrointestinal, endocrinologic, metabolic, and immunosuppressive ailments. The office of new drugs II primarily manages cardiovascular and renal drugs, urological drugs, Alzheimer drugs, and in vivo diagnostics. The office of new drugs III covers products for the peripheral and central nervous system, as well as anesthetic and analgesic drugs. The office of new drugs IV handles anti-microbial drugs, pulmonary drugs, and allergy drugs. The office of new drugs V only handles oncological drugs. The office of cellular and tissue-based products manages biosimilar products and regenerative medical products. The office of vaccine and blood products is mainly responsible for vaccines. Approved products, including drugs and regenerative medical products in Japan, are listed on the PMDA website [12,13].

## 2. Standard and Priority Reviews

Standard review and priority review designations are available in the US. The requirements for the priority review designation are as follows: It must improve safety or effectiveness significantly and be a treatment for a serious disease. As of 31 May 2019, the target total priority review time for original BLAs or NDAs (first applications of new molecular entity medical products) is eight months (six months plus a 60 calendar day for the filing review period) and six months for efficacy supplement applications. The target total standard review time for original BLAs or NDAs is 12 months (10 months plus a 60 calendar day for the filing review period) and 10 months for efficacy supplement applications (Table 1) under the Prescription Drug User Fee Act (PDUFA) V [4,14,15,16].

There are two assessment categories in the EU: The standard assessment and accelerated assessment. In the accelerated assessment and standard assessment, the CHMP must finalize its opinion within 150 and 210 days after submission of applications, respectively. However, the review time does not include the time that applicants require for responses to the CHMP’s questions [3,4,17,18]. The requirements for accelerated assessment are as follows: An importance in terms of public health and innovation, the existence of strong evidence, and the fulfillment of an unmet medical need (Table 1) [3,4,17,18]. 

In Japan, both standard review and priority review are available. Priority review is applicable to all the orphan designated products. Other than orphan drugs, the MHLW designates medical products as priority review products based on the following requirements: (1) No standard therapy exists or there is superior clinical usefulness as compared with the existing products in terms of quality of life of patients, efficacy, or safety; and (2) it applies to serious diseases [4]. As described below, drugs granted conditional approval automatically enjoy priority review designation. The target total review time for priority and standard review products is nine months and 12 months, respectively (Table 1) [4].

## 3. Orphan Designation

In the US, the Office of Orphan Products Development at the FDA may grant orphan drug designation if medical products are intended for the effective and safe treatment, prevention, or diagnosis of rare diseases affecting fewer than 200,000 people in the US, or which affect more than 200,000 people, but where the costs of marketing and developing the products are not expected to be recovered. Benefits of orphan drug designation are seven-year marketing exclusivity and financial incentives (Table 2) [19]. Orphan drug designation is not linked with accelerated approval and priority review. Medical products granted orphan designation are listed on the FDA’s website [20].

In the EU, the requirements for orphan drug designation are as follows: Developing the treatment, diagnosis, or prevention of rare diseases (fewer than five in 10,000 people in the EU); and applicable to very serious/life-threatening disease. The committee for orphan medicinal products reviews orphan designation applications. Benefits of orphan designation are ten-year exclusivity in the market and financial incentives (Table 2) [21,22]. Orphan drug designation is not linked with accelerated assessment. Medical products granted orphan designation are listed on the EMA’s website [8].

In Japan, the requirements for orphan drug designation are as follows: (1) Fewer than 50,000 patients or applicable to intractable diseases with unknown mechanisms for which standard therapy has not yet been established; (2) indicated for the treatment of serious diseases with high medical needs (where no standard therapy exists or possessing superior clinical usefulness as compared with the existing products in terms of efficacy or safety); and (3) strong rationale for the target disease and an appropriate development plan. Advantages of orphan drug designation are priority review, a 10-year reexamination period, which is similar to the market exclusivity period, and financial incentives [23] (Table 2). Therefore, medical products granted orphan drug designation automatically enjoy priority review. Lists of products designated as orphan drugs or regenerative medical products are publicly available [24,25].

## 4. Accelerated or Conditional Approval

In the US, the accelerated approval scheme was instituted in 1992. Medical products treating serious conditions, and those generally having a meaningful advantage over other available therapies, may be granted accelerated approval based on a surrogate endpoint that is reasonably likely to confer a clinical benefit. For products granted accelerated approval, postmarketing confirmatory studies are required to demonstrate the benefits. The FDA or a company may withdraw marketing authorization of products granted accelerated approval if postmarketing confirmatory clinical trials cannot demonstrate a clinical benefit, or the company cannot conduct any clinical trials that the FDA requested as a postmarketing requirement (Table 3) [14,26]. Postmarketing requirements and commitments are mentioned in the approval letter [1] and on the FDA’s main website [27]. Nagai [28] reported that 23 of the 43 solid malignancy indications and 31 of the 38 hematological malignancy indications that were granted accelerated approval were approved in the relapsed or refractory settings, and that the patient population of the postmarketing studies was different from the approved indication in 11 of 43 solid malignancy indications and 18 of 38 hematological malignancy indications; and all 18 hematological malignancy indications involved relapsed or refractory settings. In summary, accelerated approval was initially granted based on single-arm phase I or II trials for relapsed or refractory patients, and then converted to regular approval based on confirmatory phase III studies as a first-line therapy, in most cases of the drugs for hematological malignancy which were granted accelerated approval. Beaver and the FDA oncology review team [29] reported that 51 of the 93 accelerated approval indications fulfilled the postmarketing requirements and verified clinical benefit in a median of 3.4 years after their initial accelerated approval, and that 37 accelerated approval indications have not yet completed the confirmatory trials or verified clinical benefit; five indications were withdrawn from the market.

In the EU, conditional marketing authorization was instituted in 2006 and the requirements are as follows: (1) It must fulfill an unmet medical need; (2) it must be applicable to life-threatening, serious, or emergency diseases, or orphan products; (3) The company must be able to provide clinical data comprehensively; and (4) There must be a positive benefit/risk balance [4,30,31]. Conditional marketing authorization is active for one year only and annual renewal of the approval will continue until the EMA converts the conditional approval to standard authorization. In addition, conditional approval is applicable to applications of initial marketing authorization only, and efficacy supplement applications are beyond the scope of the conditional approval (Table 3). 

Marketing authorization under exceptional circumstances exists in the EU. In cases where applicants are not able to provide clinical data comprehensively because of the disease being rare, for example, marketing authorization under exceptional circumstances can be granted to medical products for life-threatening or serious diseases. In these cases, applicants do not need to submit comprehensive data in order to convert the product to normal authorization (Table 4) [4,18,31]. Marketing authorization under exceptional circumstances does not exist in the US.

In Japan, the MHLW implemented the Law on Securing Quality, Efficacy and Safety of Products including Pharmaceuticals and Medical Devices Act as the revision of the Pharmaceuticals Affairs Law in 2014 [4,9,10]. The conditional and term-limited approval system for regenerative medical products was instituted in the act. The conditional and term-limited approval is generally granted based on promising results of exploratory phase I/ II trials in terms of efficacy and safety. Sponsors must conduct postmarketing clinical studies and so on to confirm the efficacy and safety, and resubmit applications for regular approval within a predetermined period (not more than seven years) (Table 3) [32,33]. 

The conditional approval system for drugs was instituted in October, 2017 in Japan. This may be granted if all of the following requirements are met: (1) No standard therapy exists or superior clinical usefulness can be demonstrated as compared with the existing products in terms of quality of life of the patients, efficacy, or safety; (2) it is applicable to serious diseases; (3) it is difficult or it would take too long to conduct a confirmatory study; (4) exploratory clinical studies can show the efficacy and safety of the drug; and (5) surveillance or clinical studies must be conducted as a post-marketing requirement [34] (Table 4). Because these requirements include the requirements for the priority review (seriousness of the target disease and clinical usefulness of the drug), drugs granted conditional approval automatically enjoy priority review in Japan. In addition, the requirement for conditional approval, “It is difficult or it would take too long to conduct a confirmatory study” in Japan, is totally different from the accelerated approval based on a surrogate endpoint in the US. The requirement is similar to the requirement for marketing authorization under exceptional circumstances, “Companies cannot provide comprehensive clinical data because of the rarity of the disease” in the EU. Although the requirements for conditional approval for drugs in Japan include that “surveillance or clinical studies must be conducted as a post-marketing requirement,” the terms of validity for conditional approval of drugs in Japan are not established, which is different from the conditional and term-limited approval for regenerative medical products in Japan. In Japan, national insurance reimbursement is applied to regenerative medical products granted conditional and term-limited approval and drugs granted conditional approval [4]. 

As of 31 May 2019, Heart Sheet for serious heart failure by ischemic heart disease, STEMIRAC for neurological symptoms and functional disorders associated with spinal cord injury, and Collategen for chronic arterial occlusion, were granted conditional and term-limited approval for regenerative medical products in Japan [9,13,33] (Table 5). Conditional approval for all three products is valid for five or seven years (approval of Heart Sheet was exceptionally extended to eight years due to poor patient enrollment in a postmarketing study) and comparative clinical studies with an external control group are included in the postmarketing requirements for all three products. Postmarketing randomized comparative studies were not required in these cases. All the regenerative medical products granted conditional and term-limited approval, as well as all the regenerative medical products granted regular approval, except Kymriah and Collategen, were approved based on a small, single-arm clinical trial conducted only in Japan. As of 31 May 2019, only lorlatinib for anaplastic lymphoma kinase fusion-positive, non-small cell lung cancer, and pembrolizumab for microsatellite, instability-high solid cancer have been granted conditional approval for drugs in Japan [12] (Table 6). Conducting confirmatory comparative studies is not included in the postmarketing requirements for either drug. Therefore, the conditional and term-limited approval system for regenerative medical products in Japan is similar to the accelerated approval system in the US and the conditional approval system in the EU; however, the conditional approval system for drugs in Japan is only similar to the marketing authorization under exceptional circumstances system in the EU.

## 5. Breakthrough Therapy Designation 

In the US, the requirements for fast track designation are as follows: It must be a medical product for a serious disease; and the nonclinical or clinical data show its potential to address unmet needs. The features of fast track designation are a rolling review system and frequent interactions among FDA reviewers and applicants [14,35]. Fast track designation is not linked with the other expedited review programs, including priority review. The breakthrough therapy designation was instituted in 2012. The requirements for the breakthrough therapy designation are as follows: (1) The product is intended for a serious disease; and (2) preliminary clinical evidence shows a substantial improvement as compared with available therapies. Features of the designation are the FDA’s intensive guidance on a drug development program, organizational commitment involving senior management, and a rolling review system. Therefore, the advantages of breakthrough therapy designation include all the benefits of a fast track designation. Breakthrough therapy designation is not linked with the other expedited review programs, including accelerated approval [4,14,36,37]. However, it was stated that priority review could be applicable to breakthrough therapy designated products if it is suitable, and that the FDA would finish its review at least one month earlier than the PDUFA V goal date (Table 7) [14,38]. The FDA discloses only breakthrough therapy designated drugs that were granted marketing authorization [1,39]. As of June 30, 2019, eight biological medical products and 143 drugs that were granted the breakthrough therapy designation were granted marketing authorization in the US (Table 8) [39,40]. Friends of Cancer Research lists breakthrough therapy designated drugs, including those in clinical development [40]. The FDA has started the regenerative medicine advanced therapy (RMAT) designation since March, 2017 based on the 21st Century Cures Act. Requirements for the RMAT designation are as follows: (1) The drug is a regenerative medicine therapy, which is defined as a cell therapy, therapeutic tissue engineering product, human cell and tissue product, or any combination product using such therapies or products; (2) it is intended to treat, modify, reverse, or cure a serious condition; and (3) preliminary clinical evidence indicates that the regenerative medicine therapy has the potential to address unmet medical needs for the condition. Sponsors of RMAT-designated products can enjoy similar advantages to those of breakthrough therapy designation [16,41]. In addition, potential ways to support accelerated approval and satisfy post-approval requirements are addressed as the advantage of RMAT (Table 7). The number of submitted RMAT requests, granted requests, denied requests, and withdrawn requests are described on the FDA’s website, although a list of medical products granted the RMAT designation was not available as of 31 May 2019 [41].

The EMA started the PRIME (priority medicines) scheme in 2016. The PRIME scheme is limited to products under development which are not approved in the EU, and for which the sponsor intends to apply for an initial marketing authorization application through the centralized procedure via the EMA. Products eligible for the PRIME scheme are described in Table 7. These are identical to the accelerated assessment criteria in the EU. However, applicants from academia and small/medium-sized sponsors may submit a request based on nonclinical data and first-in-human studies. Advantages of the PRIME designation are shown in Table 7 [4,42,43]. The link with accelerated assessment and supporting academia and small/medium-sized sponsors are the PRIME designation’s most important features. The list of products that are currently being granted the PRIME designation on the EMA website shows that 17 of the 41 products are being developed by academia and small/medium-sized sponsors and 17 of the 41 products are ATMPs as of 31 May 2019 [42]. As of June 7, 2019, three medical products that were granted the PRIME designation were granted marketing authorization in the EU (Table 9) [42]. All of the products were ATMPs.

In Japan, the MHLW instituted, in 2015, the Sakigake (meaning pioneer or forerunner in Japanese) Designation System for medical products for diseases with unmet medical needs and that may satisfy the following two conditions: (1) The medical product was first developed in Japan, and a sponsor is planning to submit a marketing authorization application; and (2) early-phase clinical studies, non-clinical studies, and the mechanism of action suggest prominent effectiveness [4,9,33,44]. Advantages of sponsors who have medical products granted Sakigake designation are as follows: Prioritized consultation (reduced waiting time), substantial pre-application consultation, expedited review (a target total review time of six months only for drugs, devices, and IVDs), the assignment of a PMDA concierge, and an extended reexamination period (Table 7). The total review time for Sakigake designated regenerative medical products is not established. The requirement that “the medical product was first developed in Japan,” and the advantage of a specific shortened total review time are unique to the Sakigake designation. As of 31 May 2019, 22 drugs and 11 regenerative medical products have been granted Sakigake designation in Japan [45] (Table 10 and Table 11). Of these, 16 drugs and nine regenerative medical products have been developed primarily by Japanese companies. Therefore, most of the drugs and regenerative medical products granted Sakigake have been developed primarily by Japanese companies, because of the requirement that “the medical product was first developed in Japan.”

## 6. Conclusions

Several expedited regulatory review projects for innovative drugs and regenerative medical products have been developed in the US, the EU, and Japan. Each regulatory agency has elaborated an original regulatory framework and adopted the regulatory projects developed by the other regulatory agencies. For example, the FDA first developed the breakthrough therapy designation, and then the PMDA and EMA introduced the Sakigake designation and the PRIME, respectively. In addition, the necessity of the product being first development in Japan is the original feature of the Sakigake designation, while actively supporting the development of ATMPs by academia or small/medium-sized sponsors is the original feature of the PRIME designation; these particular features are different from the breakthrough therapy designation in the US. The PMDA and MHLW first developed conditional and term-limited approval for regenerative medical products in Japan and then the FDA adopted the RMAT designation in the US.

**Figure 1:**
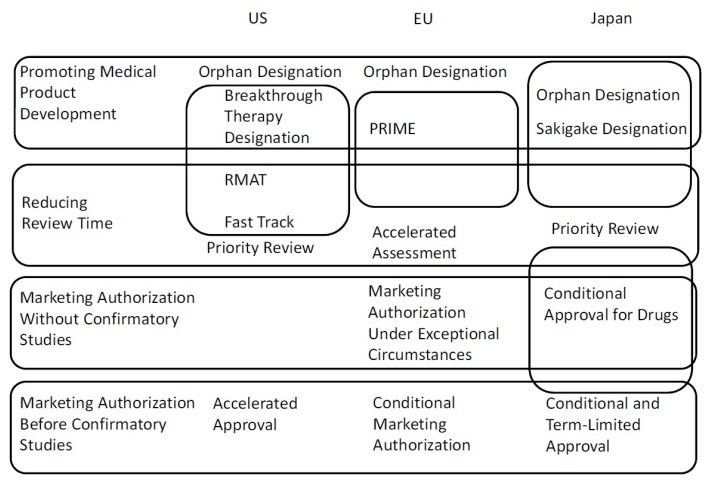
Summary of all procedures discussed in this article.

A summary of all procedures discussed in this article is described in Figure 1. As shown in Figure 1, in the US, the breakthrough therapy designation, RMAT designation, and fast track are redundant regulatory procedures. Advantages and requirements of the three procedures are complex. In the EU, the link with accelerated assessment and supporting academia and small/medium-sized sponsors are the PRIME designation’s most important features. The review time for the accelerated assessment (150 days) is shorter than that for the standard assessment by 60 days. Because the time that applicants require for responses to the CHMP’s questions is not included, however, the exact term between submission and marketing authorization is not settled. In Japan, features of conditional approval for drugs, orphan designation, and Sakigake designation include reducing review time. Moreover, the requirement for conditional approval, “It is difficult or it would take too long to conduct a confirmatory study,” in Japan, implies that drugs for rare diseases are the main scope of conditional approval. Therefore, a distinction between orphan drug designation and conditional approval for drugs in Japan is not clear.

In this review article, flexible and expedited review processes for new drugs, and cell and gene therapies in the US, the EU, and Japan are described. Moreover, all the drugs and regenerative medical products that were granted conditional approval or Sakigake designation in Japan are listed and analyzed. I believe that this article is valuable because information on up-to-date regulatory projects and approved medical products in Japan is normally only available in Japanese, making it difficult for global readers to understand and compare the Japanese regulations and approved products with those in the EU and the US.

## Figures and Tables

**Table 1:** Priority review.

	US (Priority Review)	EU (Accelerated Assessment)	Japan (Priority Review)
Features	Target total review time for original new drug applications (NDA)/biologics license applications (BLA): eight months (six months plus 60 filing days) (Standard review: 12 months (10 months plus 60 filing days)) Target total review time for efficacy supplement: six months (Standard review: 10 months)	Target total review time: 150 days (excluding the time that applicants require for responses to the CHMP’s questions) (standard assessment: 210 days)	Target total review time: nine months (standard review: 12 months)
Requirements	Significantly improves safety or effectiveness Treatment for a serious disease	Important in terms of public health and innovation, Strong evidence Fulfills an unmet medical need	No standard existing therapy or superior clinical usefulness as compared with the existing products in terms of quality of life of patients, efficacy, or safety Applicable to serious diseases or orphan drug designation

**Table 2:** Orphan drug designation.

	US	EU	Japan
Features	Financial incentives Seven-year marketing exclusivity	Financial incentives Ten-year market exclusivity	Financial incentives Ten-year market exclusivity Priority review
Requirements	Intended for the effective and safe treatment, prevention, or diagnosis of rare diseases with fewer than 200,000 people in the US; or which affect more than 200,000 people but where the costs of marketing and developing the products are not expected to be recovered	Developing the treatment, diagnosis, or prevention of rare diseases (fewer than five in 10,000 people in the EU) Very serious/life-threatening diseases	Fewer than 50,000 patients or intractable diseases with unknown mechanisms for which standard therapy has not yet been established Indicated for the treatment of serious diseases with high medical needs (no standard therapy exists or possessing superior clinical usefulness as compared with the existing products in terms of efficacy or safety) Strong rationale for the target disease and an appropriate development plan

**Table 3:** Conditional approval.

	US (Accelerated Approval)	EU (Conditional Marketing Authorization)	Japan (Conditional and Term-Limited Approval)
Features	The Food and Drug Administration (FDA) or a company may withdraw marketing authorization of products granted accelerated approval if the postmarketing confirmatory clinical trials cannot demonstrate a clinical benefit or the company cannot conduct any clinical trials that the FDA requested as a postmarketing requirement	Applicable to applications of initial marketing authorization only, with efficacy supplement applications being beyond the scope Active for one year only with an annual renewal of the approval continuing until the European Medicines Agency (EMA) converts the conditional approval to standard authorization	Only for regenerative medical products Valid for no more than seven years
Requirements	Drugs treating serious conditions Demonstrating a meaningful advantage over other available drugs based on a surrogate endpoint that is reasonably likely to infer a clinical benefit Postmarketing confirmatory studies are required to demonstrate benefits	Fulfilling an unmet medical need Pertaining to life-threatening, serious, or emergency diseases, or orphan products The company being able to provide clinical data comprehensively A positive benefit/risk balance	Promising results of early-phase phase I/II registration trials in terms of efficacy and safety Sponsors must conduct postmarketing clinical studies and so on to confirm the efficacy and safety and resubmit applications for regular approval within a predetermined period (no more than seven years) (recent examples indicate that postmarketing randomized comparative studies are not necessary and postmarketing comparative clinical studies with external control group are acceptable)

**Table 4:** Marketing authorization under exceptional circumstances.

	US (None)	EU (Marketing Authorization Under Exceptional Circumstances)	Japan (Conditional Approval)
Features	-	Applicants do not need to submit comprehensive data in order to convert to normal authorization	Conditional approval for drugs Priority review
Requirements	-	Applicants are not able to provide clinical data comprehensively because of the rarity of the disease for example Applicable to life-threatening or serious diseases	No standard therapy exists or superior clinical usefulness is demonstrated as compared with the existing products in terms of quality of life of patients, efficacy, or safety Applicable to serious diseases It is difficult or it would take too long to conduct a confirmatory study Exploratory clinical studies show efficacy and safety Surveillance or clinical studies must be conducted as a post-marketing requirement (recent examples indicate that postmarketing comparative studies are not necessary and postmarketing surveillance is acceptable)

**Table 5:** Regenerative medical products granted approval in Japan as of 31 May 2019.

Brand Name (Company)	Non-proprietary Name	Indication	Approval Date	Number of Enrolled Patients (Japanese)	Primary Endpoint	Post-Marketing Requirement
JACE (Japan Tissue Engineering)	Human Autologous Epidermis-derived Cell Sheet	Serious and Extensive Burns	29 October 2007(Regular Approval) Priority Review	2 (2) Single-arm	Epidermal Replacement Rate at Four Weeks	Epidermal replacement rate at four weeks in a single-arm study with 30 patients (Completed)
JACC (Japan Tissue Engineering)	Human Autologous Cartilage Cells	Traumatic Cartilage Defects	27 July 2012 (Regular Approval)	33 (33) Single-arm	Improvement in Function and Cartilage Repair at 12 Months	None (Post-marketing Surveillance Only)
TEMCELL HS (JCR Pharma)	Human Allogeneic Bone Marrow-derived Mesenchymal Stem Cells	Acute Graft Versus Host Disease	18 September 2015 (Regular Approval) Orphan Designation	25 (25) Single-arm	Complete Response Rate with ≥28 Days Duration	None (Post-marketing Surveillance Only)
Heart Sheet (Terumo)	Human Autologous Skeletal Myoblast -derived Cell Sheet	Serious Heart Failure by Ischemic Heart Disease	18 September 2015 (Conditional and Term-Limited Approval: five years -> eight years)	7 (7) Single-arm	Left Ventricular Ejection Fraction at 26 Weeks	Compare time to cardiac event-related death prospectively between 60 patients treated with Heart Sheet and 120 patients with severe heart failure who are potentially eligible for Heart Sheet but are receiving other treatment as the external control group (Ongoing)
JACE (Japan Tissue Engineering)	Human Autologous Epidermis-derived Cell Sheet	Giant Congenital Melanocytic Nevus	29 September 2016 (Regular Approval) Orphan Designation	11 (11) Single-arm	≥95% Epithelialization of Grafted Area at 12 Weeks	None (Post-marketing Surveillance Only)
JACE (Japan Tissue Engineering)	Human Autologous Epidermis-derived Cell Sheet	Dystrophic Epidermolysis Bullosa and Junctional Epidermolysis Bullosa	28 December 2018 (Regular Approval) Orphan Designation	4(4) Single-arm 3 (3) Single-arm	Epidermal Replacement Rate at four Weeks	None (Post-marketing Surveillance Only)
STEMIRAC (Nipuro)	Human Autologous Bone Marrow-derived Mesenchymal Stem Cell	Neurological Symptoms and Functional Disorders Associated with Spinal Cord Injury	28 December 2018 (Conditional and Term-Limited Approval: 7 years) Sakigake Designation	17 (17) Single-arm	Improvement in American Spinal Injury Association Impairment Scale at 220 Days	Compare improvement in American Spinal Injury Association Impairment Scale prospectively between 198 patients treated with STEMIRAC and 414 patients with spinal cord injury who are potentially eligible for STEMIRAC but are receiving other treatment as the external control group (Ongoing or Not Started)
Kymriah (Novartis)	Tisagenlecleucel (CD19 CAR T-cell)	Relapsed and Refractory CD19-positive B-cell Acute Lymphoblastic Leukemia (B-ALL) Relapsed and Refractory CD19-positive Diffuse Large B-cell Lymphoma (DLBCL)	26 March 2019 (Regular Approval) Orphan Designation	B-ALL: 92 (6) Single-arm DLBCL: 165 (9) Single-arm	B-ALL: Complete Response Rate DLBCL: Overall Response Rate	None (Post-marketing Surveillance Only)
Collategen (AnGes)	Beperminogene Perplasmid (Hepatocyte Growth Factor Plasmid)	Chronic Arterial Occlusion (Arteriosclerosis Obliterans and Buerger’s Disease)	26 March 2019 (Conditional and Term-Limited Approval: five years)	Arteriosclerosis Obliterans: 44 (44) Comparative Collategen: 29, Placebo: 15 Buerger’s Disease: 10 (10) Single-arm Arteriosclerosis Obliterans 2 (2), Buerger’s Disease 4 (4) Single-arm	Arteriosclerosis Obliterans: Improvement in Pain Visual Analogue Scale or Ulcer Size at 12 Weeks Buerger’s Disease: Improvement in Ulcer Size at 12 Weeks Improvement in Pain Visual Analogue Scale or Ulcer Size at 12 Weeks	Compare complete occlusion of ulcer rate prospectively between 120 patients treated with Collategen and 80 patients with Arteriosclerosis Obliterans and Buerger’s Disease who are potentially eligible for Collategen but are receiving other treatment as the external control group (Ongoing or Not Started)

**Table 6:** Drugs granted conditional approval in Japan as of 31 May 2019.

Drug	Indication	Approval Date	Number of Enrolled Patients (Japanese)	Primary Endpoint	Post-marketing Requirement
Lorlatinib	Anaplastic Lymphoma Kinase fusion-positive Non-Small Cell Lung Cancer	21 September 2018	197 (31) in one phase 2 study	Overall Response Rate	Post-marketing surveillance
Pembrolizumab	Microsatellite Instability-high solid cancer	21 December 2018	155 (14) in two phase 2 studies	Overall Response Rate	Post-marketing surveillance Final analysis of the ongoing two phase 2 studies

**Table 7:** Breakthrough therapy designation.

	US (Breakthrough Therapy Designation or Regenerative Medicine Advanced Therapy (RMAT) Designation)	EU Priority Medicines (PRIME)	Japan (Sakigake)
Features	Rolling review (Fast Track Designation) FDA’s intensive guidance on a drug development program Organizational commitment involving senior management Priority review if applicable, with the FDA finishing its review at least one month earlier than the Prescription Drug User Fee Act (PDUFA) V goal date (Help with addressing potential ways to support accelerated approval and satisfy post-approval requirements only for RMAT)	The appointment of a rapporteur from the Committee for Medicinal Products for Human Use (CHMP) or the Committee for Advanced Therapies (CAT) to provide continuous support The organization of a kick-off meeting with the rapporteur and experts to provide guidance on development plan and regulatory strategy The assignment of a dedicated point of contact The provision of scientific advice at key development milestones Potential for accelerated assessment	Prioritized consultation (reduced waiting time) Substantial pre-application consultation Expedited review (a target total review time of six months for drugs, devices, and IVDs, and a designated priority review) (the total review time for Sakigake -designated regenerative medical products is not established) Assignment of a Pharmaceuticals and Medical Devices Agency (PMDA) concierge An extended reexamination period
Requirements	(Breakthrough Therapy Designation) It is intended to treat a serious condition Preliminary clinical evidence indicates that the drug demonstrates substantial improvements on a clinically significant endpoint over available therapies (RMAT) The drug is a regenerative medicine therapy, which is defined as a cell therapy, therapeutic tissue engineering product, human cell and tissue product, or any combination product using such therapies or products It is intended to treat, modify, reverse, or cure a serious condition The preliminary clinical evidence indicates that the regenerative medicine therapy has the potential to address unmet medical needs for the condition	Products under development are not approved in the EU and for which a sponsor intends to apply for an initial marketing authorization application through the centralized procedure via the EMA It targets conditions with an unmet medical need (no satisfactory method of diagnosis, prevention, or treatment, or is of major therapeutic advantage to patients) (identical to the accelerated assessment criteria) It demonstrates the potential to address the unmet medical need in terms of maintaining and improving the health and is of major public health interest from the viewpoint of therapeutic innovation (identical to the accelerated assessment criteria)	Medical products for diseases with unmet medical need The medical product has been first developed in Japan, and a sponsor is planning to submit a marketing authorization application Early-phase clinical studies, non-clinical studies, and the mechanism of action suggest prominent effectiveness

**Table 8:** Biological medical products that were granted the breakthrough therapy designation, which were granted marketing authorization in the US as of 30 June 2019.

Drug	Indication	Date of Granting the Breakthrough Therapy Designation Disclosure	Approval Date
Meningococcal Group B Vaccine	Active immunization to prevent invasive meningococcal disease caused by N. meningitidis serogroup B in individuals 10 through 25 years of age	20 March 2014	29 October 2014
Meningococcal Group B Vaccine	Active immunization to prevent invasive meningococcal disease caused by N. meningitidis serogroup B in individuals 10 through 25 years of age	7 April 2014	23 January 2015
Tisagenlecleucel	For the treatment of pediatric and young adult patients (age 3-25 years) with B-cell precursor acute lymphoblastic leukemia that is refractory or in second or later relapse.	7 July 2014	30 August 2017
Axicabtagene Ciloleucel	Treatment of adult patients with relapsed or refractory large B-cell lymphoma after two or more lines of systemic therapy, including diffuse large B-cell lymphoma (DLBCL) not otherwise specified, primary mediastinal large B-cell lymphoma, high grade B-cell lymphoma, and DLBCL arising from follicular lymphoma. Axicabtagene ciloleucel is not indicated for the treatment of patients with primary central nervous system lymphoma.	7 December 2015	18 October 2017
Voretigene Neparvovec	The treatment of patients with confirmed biallelic RPE65 mutation-associated retinal dystrophy.	24 September 2014	19 December 2017
Tisagenlecleucel	For the treatment of adult patients with relapsed or refractory diffuse large B-cell lymphoma who are ineligible for autologous transplant.	18 April 2017	1 May 2018
Coagulation Factor Xa (Recombinant), Inactivated-zhzo	Indicated for patients treated with rivaroxaban and apixaban, when reversal of anticoagulation is needed due to life-threatening or uncontrolled bleeding.	25 November 2013	3 May 2018
Onasemnogene abeparvovec	Indication-treatment of pediatric patients with spinal muscular atrophy (SMA) Type 1 with or without disease onset.	20 July 2016	24 May 2019

**Table 9:** Medical products that were granted the priority medicine (PRIME) designation that were granted marketing authorization in the EU as of 7 June 2019.

Drug	Indication	Date of Granting the PRIME	Approval Date
Tisagenlecleucel	Treatment of pediatric patients with relapsed or refractory B cell acute lymphoblastic leukemia	23 June 2016	22 August 2018
Axicabtagene ciloleucel	Treatment of adult patients with diffuse large B-cell lymphoma who have not responded to their prior therapy, or have had disease progression after autologous stem cell transplant	26 May 2016	23 August 2018
Autologous CD34+ hematopoietic stem cells transduced with lentiviral vector encoding the human βA-T87Q-globin gene (Lentiglobin)	Treatment of transfusion-dependent beta-thalassemia	15 September 2016	29 May 2019

**Table 10:** Sakigake-designated drugs in Japan as of 31 May 2019.

Non-Proprietary Name (Company)	Country Where the Head Quarter Office of the Company That Is Primarily Developing the Product Exists	Characteristics	Indication	Designation Date	Marketing Authorization
Sirolimus (Nobelpharma)	Japan	Mammalian Target of Rapamycin inhibitor	Skin lesions associated with tuberous sclerosis complex	27 October 2015	Approved on 23 March 2017
NS-065/NCNP01 (Nippon Shinyaku)	Japan	Exon 53 skipping of a dystrophin mRNA	Duchenne Muscle Dystrophy	27 October 2015	No
Baloxavir marboxil (Shionogi)	Japan	Cap-dependent endonuclease inhibitor	Influenza A or B virus infections	27 October 2015	Approved on 23 February 2018
BCX7353 (BioCyst Pharmaceuticals -> Integrated Development Associates)	Foreign	Kallikrein inhibitor	Hereditary Angioedema	27 October 2015	No
Gilteritinib fumarate (Astellas Pharma)	Japan	FLT3 inhibitor	Relapsed or refractory FLT3 mutation-positive acute myeloid leukemia	27 October 2015	Approved on 21 September 2018
Pembrolizumab (MSD)	Foreign	Anti-PD 1 antibody	Advanced Gastric Cancer	27 October 2015 Currently declined	No
Olipudase Alfa (Sanofi)	Foreign	Recombinant human acid sphingomyelinase	Acid sphingomyelinase deficiency	21 April 2017	No
Aducanumab (Biogen)	Foreign	Anti-Amyloid beta antibody	Alzheimer’s Disease	21 April 2017	No
DS-5141b (DAIICHI SANKYO)	Japan	Exon 45 skipping of a dystrophin mRNA	Duchenne muscular dystrophy	21 April 2017	No
SPM-011 (STELLA PHARMA)	Japan	Boron Neutron Capture Therapy	Recurrent malignant glioma, unresectable local recurred head and neck cancer, and advanced non squamous cell carcinoma	21 April 2017	No
Nivolumab (ONO PHARMACEUTICAL)	Japan	Anti-PD 1 antibody	Cholangiocarcinoma	21 April 2017	No
RTA402 (Kyowa Kirin)	Japan	Nrf2 activator	Diabetic Nephropathy	27 March 2018	No
JR-141 (JCR Pharma)	Japan	Anti-transferin receptor antibody-fused iduronate-2-sulfatase	Mucopolysaccharidosis type II (Hunter syndrome)	27 March 2018	No
tafamidis meglumine (Pfizer)	Foreign	Transthyretin stabilizer	Transthyretin-mediated amyloidosis	27 March 2018	Approved on 26 March 2019
MSC2156119J (Merck Biopharma)	Foreign	c-MET inhibitor	MET exon 14 skipping-positive NSCLC	27 March 2018	No
Trastuzumab deruxtecan (DAIICHI SANKYO)	Japan	Anti-HER2 antibody-drug conjugate	Relapsed HER2-positive gastric cancer	27 March 2018	No
Entrectinib (Chugai Pharmaceutical)	Japan	TRK inhibitor	Adult and pediatric NTRK fusion gene-positive solid cancer	27 March 2018	No
Valemetostat (DAIICHI SANKYO)	Japan	EZH 1/2 inhibitor	Relapsed and refractory peripheral T-cell lymphoma	8 April 2019	No
Ixazomib (Takeda Pharmaceutical)	Japan	Proteasome inhibitor	AL amyloidosis	8 April 2019	No
TAK-925 (Takeda Pharmaceutical)	Japan	Orexin 2 Receptor-selective agonist	Narcolepsy	8 April 2019	No
ASP-1929 (Rakuten Medical)	Japan	Cetuximab and IRDye 700DX activated with red light using a proprietary investigational laser and fiber optics	Head and neck cancer	8 April 2019	No
E7090 (Eisai)	Japan	FGFR 1/2/3 inhibitor	FGFR2 fusion gene-positive Cholangiocarcinoma	8 April 2019	No

**Table 11:** Sakigake-designated regenerative medical products in Japan as of 31 May 2019.

Product Name (Company)	Country where the Head Quarter Office of the Company That Is Primarily Developing the Product Exists	Characteristics	Indication	Designation Date
STEMIRAC (Nipuro)	Japan	Human Autologous Bone Marrow-derived Mesenchymal Stem Cell	Neurological Symptoms and Functional Disorders Associated with Spinal Cord Injury	10 February 2016
G47 Delta (DaiichiSankyo)	Japan	Oncolytic Herpes Simplex Virus-1	Malignant Glioma	10 February 2016
JRM-001 (Japan Regenerative Medicine)	Japan	Human Autologous Cardiac Progenitor/Stem Cells	Pediatric Congenital Heart Disease (Single Ventricle Physiology)	10 February 2016
CLS2702C/D (CellSeed)	Japan	Human Autologous Oral Mucosal Epithelial Cell Sheet	Prevention of Esophageal Partial Narrowing after Endoscopic Submucosal Dissection	28 February 2017
None (Sumitomo Dainippon Pharma)	Japan	Human Allogeneic iPS-derived Dopamine Neural Progenitor Cells	Parkinson’s Disease	28 February 2017
HLCM051 (Healios)	Japan	Human Allogeneic Bone Marrow Progenitor/Stem Cells	Cerebral Infarction	28 February 2017
TBI-1301 (Takara Bio and Otsuka Pharmaceutical)	Japan	NY-ESO-1 siTCR Gene Therapy	Synovial Sarcoma	27 March 2018
CLBS12 (Caladrius Biosciences)	Foreign	Human Autologous CD34-positive Peripheral Blood Cells	Critical Limb Ischemia	27 March 2018
AVXS-101 (AveXis and Novartis)	Foreign	Human SMN Adeno-associated Virus 9 Gene Therapy	Spinal Muscular Atrophy	27 March 2018
OBP-301 (Oncolys BioPharma)	Japan	hTERT Promotor Oncolytic Adenovirus	Esophageal Cancer	8 April 2019
SB623 (SanBio)	Japan	Adult Allogeneic Bone Marrow-derived Mesenchymal Stem Cells	Chronic Motor Deficit Resulting from Traumatic Brain Injury	8 April 2019

## Abbreviations

IVD	in vitro diagnostics
FDA	Food and Drug Administration
NDA	new drug applications
BLA	biologics license applications
EMA	European Medicines Agency
ATMP	advanced-therapy medicinal products
CHMP	Committee for Medicinal Products for Human Use
CAT	Committee for Advanced Therapies
PMDA	Pharmaceuticals and Medical Devices Agency
MHLW	Ministry of Health, Labour and Welfare
PDUFA	Prescription Drug User Fee Act
RMAT	regenerative medicine advanced therapy
PRIME	priority medicines
